# Green Synthesized Silver Nanoparticles: A Potential Antibacterial Agent, Antioxidant, and Colorimetric Nanoprobe for the Detection of Hg^2+^ Ions

**DOI:** 10.1002/gch2.202300072

**Published:** 2023-07-20

**Authors:** Prianka Saha, Md. Morsaline Billah, A. B. M. Nazmul Islam, Md. Ahsan Habib, Md. Mahiuddin

**Affiliations:** ^1^ Chemistry Discipline Khulna University Khulna 9208 Bangladesh; ^2^ Biotechnology and Genetic Engineering Discipline Khulna University Khulna 9208 Bangladesh

**Keywords:** antibacterial agent, antioxidant, mercury detection, nanoprobe, silver nanoparticles

## Abstract

Silver nanoparticles (AgNPs) prepared by green synthesis have a lot of potentials in various fields. Among them, as an antioxidant, antibacterial agent, and nanoprobe for the colorimetric detection of mercury (Hg^2+^) ions is thought to be the most important. The antibacterial, antioxidant, and colorimetric sensing potential of the greenly produced AgNPs utilizing *Piper chaba* stem extract are all predicted in this investigation. By using the disc diffusion method, the antibacterial activity of greenly produced AgNPs are assessed, and the findings are measured from the zone of inhibition (ZOI). It is revealed that the *Staphylococcus aureus, Micrococcus spp., Escherichia coli*, and *Pseudomonas aeruginosa* bacterial strains are significantly resisted by the greenly produced AgNPs. The antioxidant activity test of AgNPs reveals a considerable impact on free radical scavenging having the inhibitory concentration (*IC*_50_) is 1.13 mL (equivalent to 0.45 mg mL^−1^). Also, with a low limit of detection of 28 ppm, the resulting AgNPs are used as highly selective and economical colorimetric sensors for Hg^2+^ detection. The study's findings support the hypothesis that *Piper chaba* stems can serve as a source for the production of AgNPs with high antibacterial and antioxidant activity and usefulness for simple colorimetric readings of Hg^2+^.

## Graphical abstract

*Piper chaba* stem extract‐mediated green synthesized silver nanoparticles have excellent stability and act as potential antibacterial agents against different bacterial strains and as colorimetric nanoprobe for the detection of mercury (II) ions, in addition to possessing strong antioxidant capabilities.

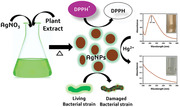

## 1 Introduction

Nanoparticles (NPs) have attained a lot of attention from scientists due to their notable or exceptionally improved properties depending on certain parameters, such as size, shape, distribution, and morphology. Metal NPs distinctive physicochemical characteristics, including their large surface area, high reactivity, large surface‐to‐volume ratio, spatial confinement, tunable size, and shape establish the bridge to overcome the gap between the bulk and the atomic structures in terms of their attributes. An effective study is done on the NPs made using noble metals, especially gold, silver, platinum, and palladium.^[^ ^1^, ^2^, ^3^ ^]^

Due to their distinct physicochemical characteristics, silver nanoparticles (AgNPs) have received the most attention of all the existing nanomaterial types.^[^ ^3^, ^4^ ^]^ AgNPs have numerous applications, such as in optics,^[^ ^5^, ^6^ ^]^ selective coating,^[^ ^7^ ^]^ water purification,^[^ ^8^ ^]^ catalysis,^[^ ^9^, ^10^, ^11^, ^12^, ^13^ ^]^ biolabeling,^[^ ^14^ ^]^ biomedicine,^[^ ^15^ ^]^ food chemistry,^[^ ^16^ ^]^ agriculture,^[^ ^17^ ^]^ cosmetics,^[^ ^18^ ^]^ food packaging,^[^ ^19^ ^]^ and sensing.^[^ ^5^, ^20^, ^21^, ^22^, ^23^, ^24^ ^]^ They are extensively researched as antioxidant,^[^ ^25^, ^26^, ^27^, ^28^, ^29^ ^]^ antibacterial,^[^ ^25^, ^26^, ^30^, ^31^, ^32^ ^]^ antifungal,^[^ ^33^, ^34^ ^]^ anti‐inflammatory,^[^ ^35^ ^]^ antiviral,^[^ ^36^ ^]^ and anticancer substances^[^ ^25^, ^26^, ^37^, ^38^ ^]^ in the field of pharmacology. AgNPs are widely known for their potent antibacterial activity^[^ ^8^, ^39^, ^40^, ^41^ ^]^ and their incredibly cost‐effective and selective colorimetric sensor for the detection of metal ions.^[^ ^22^, ^24^, ^42^, ^43^, ^44^ ^]^

Several studies have been undertaken and found that the green synthesis of AgNPs from plant sources could be a promising and biocompatible substitute for one involving conventional chemicals. Among wide range of applications, the greenly synthesized AgNPs have shown excellent potential as a promising nanoprobe for the highly selective detection of Hg^2+^, a powerful broad‐spectrum antibacterial agent, and an outstanding antioxidant.^[^ ^22^, ^23^, ^25^, ^44^, ^45^ ^]^ This study has also been centered on these applications.

The antibacterial activity of AgNPs is crucial in the sectors of agriculture, food, medicine, and the environment. The physicochemical characteristics of plant extract mediated synthesized AgNPs, such as stability, size, shape, and surface charge, as well as capping agents, have a significant impact on their antibacterial effectiveness.^[^ ^46^ ^]^ It is thought that these qualities are essential for antibacterial action. In addition to serving as agglomerators of the nanoparticles, capping compounds such as flavonoids, alkaloids, terpenoids, and steroids significantly enhance the antibacterial activity of AgNPs. Hence, it is essential to investigate plant extracts that include biomolecules that have inherent antibacterial activity in order to synthesize AgNPs with a rational antibacterial activity.^[^ ^2^, ^3^, ^25^, ^30^, ^47^, ^48^, ^49^, ^50^, ^51^, ^52^ ^]^

A well‐known hazardous element that has detrimental effects on both human health and the environment is mercury (Hg). Hg^2+^ ions have been widely detected using a variety of sensing techniques, such as atomic absorption spectrometry (AAS), atomic emission spectrometry (AES), and inductively coupled plasma mass spectrometry (ICP‐MS). These technologies, however, have limited uses for quick and in‐field investigation because they call for expensive equipment and complicated procedures. To detect Hg^2+^, the development of biomaterials‐based colorimetric sensors has been carried out to solve these disadvantages. The label‐free AgNPs‐based colorimetric sensors for Hg^2+^ ions in water have recently come under increasing scrutiny due to their inherent high sensitivity, low detection limit, and simple colorimetric read‐out.^[^ ^22^, ^24^, ^43^, ^53^ ^]^ In these colorimetric assays, piperine and other organic compounds with functional groups like amine, amide, hydroxy, and hetero‐aromatic rings functionalize the biosynthesized AgNPs. And via electrostatic interactions or hydrogen bonding, these compounds can either assemble or stabilize AgNPs. Interestingly, AgNPs‐based colorimetric sensors for Hg^2+^ ion detection result from interactions between nanoparticles and Hg^2+^, which cause nanoparticles to aggregate or crosslink or oxidation‐reduction among themselves and provide distinctive colorimetric responses.^[^ ^1^, ^3^, ^22^, ^23^, ^42^, ^54^, ^55^, ^56^ ^]^ There have been no studies on the use of *Piper chaba*‐based greenly synthesized AgNPs for antibacterial, antioxidant action, or very sensitive colorimetric Hg^2+^ detection up to this point.

As a reducing and stabilizing agent, *Piper chaba* stem extract was used in a green synthesis of AgNPs that we reported previously. The resulting AgNPs efficiently catalyzed the reduction of 4‐nitrophenol (4‐NP) and methylene blue (MB) in the presence of sodium borohydride, and they are capped with the phytochemicals, especially containing amides and hydroxyl functionalities and they displayed outstanding colloidal dispersibility.^[^ ^57^ ^]^ We presume that the *Piper chaba* stem extract‐mediated green synthesized AgNPs will be exhibited similar characteristics to other green synthesized AgNPs, including strong antioxidant and antibacterial action as well as good selectivity for the detection of Hg^2+^.

In this study, we have shown how to use greenly prepared AgNPs, which are mediated by *Piper chaba* stem extract, as an effective antibacterial agent against both gram‐positive and gram‐negative bacterial strains, as well as a potential nanoprobe for the colorimetric detection of Hg^2+^. The study also focused on the use of 2,2‐diphenyl‐1‐picrylhydrazyl (DPPH) free radical‐scavenging assay to estimate the antioxidant activity of greenly prepared AgNPs.

## 2 Results and Discussion

### 2.1 Formation of AgNPs

**Figure 1:**
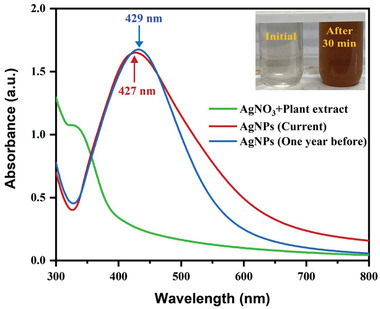
Optical images of the reaction mixtures before and after the reaction with the UV–vis spectrum of AgNPs prepared currently and a year earlier from AgNO_3_ and *Piper chaba* stem extract.

**Figure 2:**
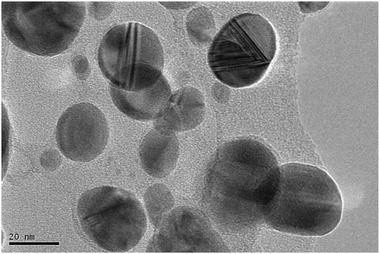
TEM image of AgNPs obtained from AgNO_3_ and *Piper chaba* stem extract.

First, visual observation was put to use to observe the formation of AgNPs using *Piper chaba* stem extract. Similar to the previous report, the change of color from colorless to reddish‐brown confirms the formation of AgNPs. The formation of AgNPs was further confirmed by its characteristics surface plasmon resonance (SPR) band from the UV–vis spectrum at ≈427 nm (**Figure** **1**). The resulting AgNPs dispersed stably over 1 year without any aggregation and precipitation. To verify the stability, we nonetheless included the AgNPs UV spectrum that had been synthesized a year earlier to Figure 1, which have a similar SPR band with a negligible redshift to the AgNPs that are currently being produced. The transmission electron microscopy (TEM) image of the AgNPs (**Figure** **2**) shows that the particles are spherical with an average size of 20 nm. These results are almost identical to the TEM image shown in the previous report.^[^ ^57^ ^]^

### 2.2 Antibacterial Activity Test

**Figure 3:**
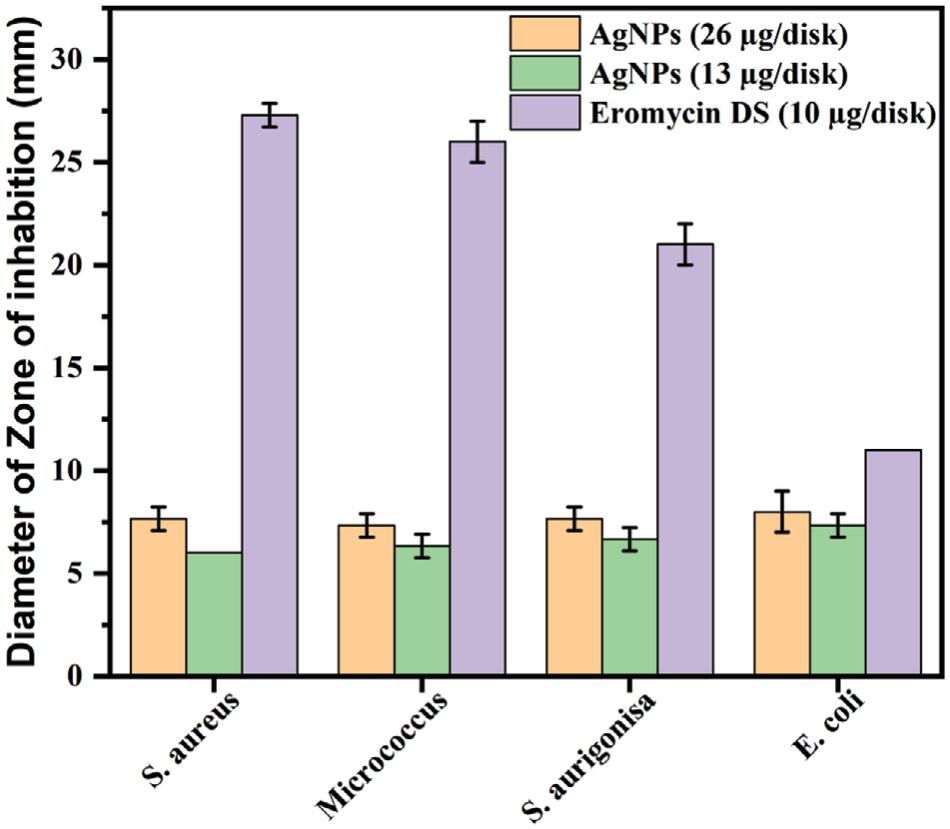
Zone of inhibition of green synthesized AgNPs against various bacterial strains, measured in diameter.

The disc diffusion method is largely approved for the preliminary screening of antibacterial activity. In this experiment, we looked at the antibacterial effectiveness of two different concentrations of greenly synthesized AgNPs (26 and 13 µg disc^−1^). Eromycin DS (10 µg disc^−1^) was used as a standard antibiotic disc for comparison purposes. The finding of the disk diffusion test is summarized in **Figure** **3**. In this test, the presence of a clear zone of inhibition around the AgNPs disk suggests that the AgNPs showed antibacterial activity which can inhibit the growth of human pathogenic gram‐positive and gram‐negative bacteria. Moreover, when the concentration of AgNPs increased, the zone of inhibition grew. Small, nonaggregated AgNPs with a large specific surface area can easily penetrate bacterial cell walls and nuclear contents and attach to the proteins that weaken cell walls. Additionally, as *Piper chaba* is widely known for its antibacterial action,^[^ ^58^, ^59^ ^]^ the association of the phytochemicals and the activity of AgNPs may be amplified through a synergistic effect.

Against each of the investigated bacterial strains, the AgNPs solution exhibits significant antibacterial activity. This activity of AgNPs may offer valuable applications in various fields, such as, in the food packaging materials and in the medical devices.

### 2.3 Antioxidant Activity Test

**Figure 4:**
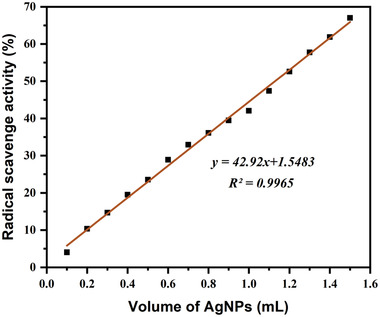
The relationship between scavenging activity (%) and AgNPs volume (mL) for the green synthesized AgNPs in the DPPH scavenging Assay.

The antioxidant assay is an essential area for biologists and phytochemists. The antioxidant efficacy of AgNPs was quantified spectrophotometrically by observing the DPPH radical color from purple to yellow. The DPPH radical scavenging activity of green synthesized AgNPs using *Piper chaba* stem extract was presented in **Figure** **4**, which shows a good linear relationship between scavenging activity and volume of AgNPs, and found that the DPPH radical activity of AgNPs was increasing from 0.1 to 1.5 mL. At concentrations, of 0.1 to 1.5 mL, AgNPs showed a scavenging rate ranging from 4% to 67%. The 50% scavenging efficacy was found at 1.13 mL (equivalent to 0.45 mg mL^−1^) of AgNPs water dispersion. This study reveals that the antioxidant activity of AgNPs is showing increasing trend with increasing the volume of silver nanoparticles solution. Therefore, in the quantitative antioxidant assay, AgNPs revealed DPPH free radical scavenging activity with an approximate *IC*_50_ value of 1.13 mL (equivalent to 0.45 mg mL^−1^) which is noteworthy.

### 2.4 Hg^2+^ Detection Capacity of Synthesized AgNPs

#### 2.4.1 Selectivity of AgNPs

**Figure 5:**
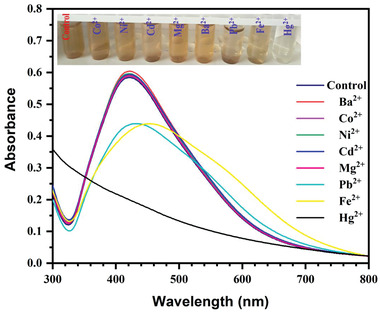
Absorption spectra of AgNPs when exposed to various metal ions (illustration: photo images of corresponding samples).

**Figure 6:**
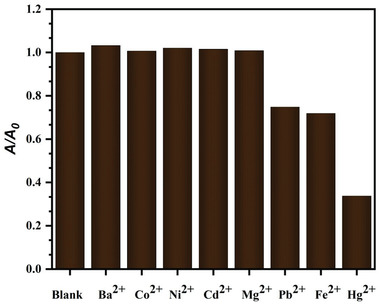
The relative histogram of absorbance ratio (*A/A*_0_) of green synthesized AgNPs with various metal ions.

For the analysis of AgNPs performance as a colorimetric probe, the most important factor is the selectivity test. Surface Plasmon Resonance (SPR) absorbance measurements were used to study the colorimetric reactions of AgNPs to the different cations Ba^2+^, Co^2+^, Cd^2+^, Fe^2+^, Hg^2+^, Mg^2+^, Ni^2+^, and Pb^2+^ individually. The characteristic peak of AgNPs is located at 427 nm as observed in **Figure** **5**. The SPR of AgNPs with all samples was thoroughly compared. To perform this, 70 ppm of each cation was mixed with an equal amount of AgNPs individually, for most of the cations, the intensity of the absorbance and peak position remain unchanged, indicating that the state of the AgNPs was unaffected. The SPR of AgNPs, however, changed noticeably when exposed to Fe^2+^, Pb^2+^, and Hg^2+^ (Figure 5), and in the case of Hg^2+^, the absorbance of AgNPs nearly vanished, which showed a significant difference to other ions. And related optical images demonstrated the amazing color change. Figure 5 shows the different colorimetric reactions to the Hg^2+^ (brown to colorless) (inset). In contrast, the AgNPs containing other ions developed the same natural color as AgNPs. Both the colorimetric response (**Figure** **6**) and the AgNPs absorbance intensity with all other ions intuitively reflected the strong contrast to the Hg^2+^ sample, indicating the presence of significant Hg^2+^ aggregation, and all these outcomes indicated the AgNPs colorimetric nanoprobe offered selectivity for Hg^2+^ detection.

**Figure 7:**
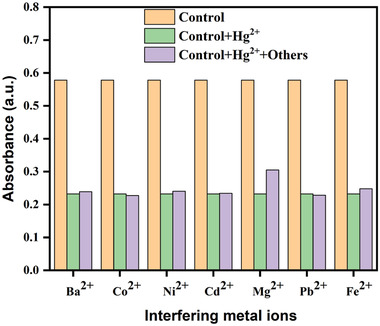
The relative histogram of absorbance of green synthesized AgNPs with various interfering metal ions.

**Figure 8:**
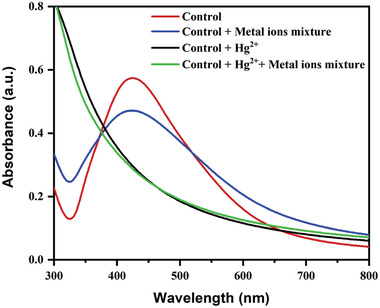
Absorption spectra of AgNPs when exposed to the mixture of various metal ions.

Aqueous solutions containing 35 ppm of the following metal ions were prepared: Ba^2+^, Co^2+^, Ni^2+^, Cd^2+^, Mg^2+^, Pb^2+^, Fe^2+^, and Hg^2+^. The total volume of the mixture (4 mL) was kept constant while mixing an equal proportion of AgNPs, Hg^2+^, and interfering metals solutions. AgNPs and Hg^2+^ solutions turned colorless after 1 min, and the SPR band of AgNPs decreased with a same trend, while there were interfering metal ions present (**Figure** **7**). In addition, was noticed that even with all of the interfering metal ions present, the AgNPs and Hg^2+^ solutions became colorless and the SPR band of AgNPs completely disappeared (**Figure** **8**). According to the aforementioned finding, it is easy to articulate that *Piper chaba* stem extract‐mediated green synthesized AgNPs can detect Hg^2+^ ions with high specificity even when there are equimolar amounts of other interfering cations from other metals present. Similar results were also observed in various reports.^[^ ^1^, ^23^, ^54^, ^60^ ^]^

#### 2.4.2 Sensitivity

**Figure 9:**
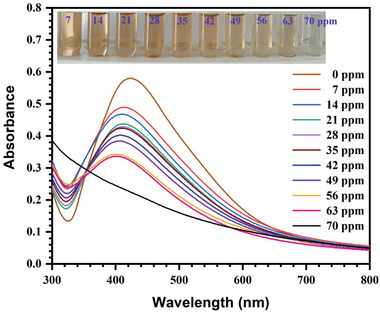
UV–vis absorption spectra of the mixtures of AgNPs and Hg^2+^ with varied Hg^2+^ concentrations (7–70 ppm) (inset: picture of color changes of the mixtures as a function of Hg^2+^ concentration).

**Figure 10:**
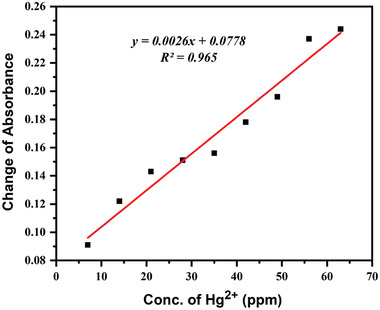
A plot illustrating the regression coefficient between the change in absorbance and the concentration of Hg^2+^.

**Figure 11:**
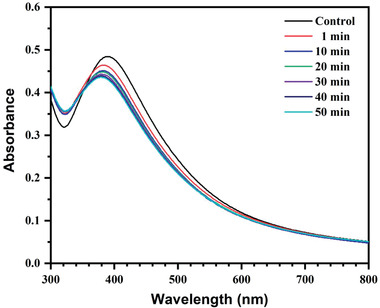
Time‐dependent UV–vis spectra of AgNPs dispersion in the presence of Hg^2+^ ions (35 ppm).

The impact of the various ion concentrations on the absorbance was carefully assessed to quantitatively ensure the performance for Hg^2+^ detection utilizing AgNPs as a colorimetric probe. **Figure** **9** shows the SPR absorbance of AgNPs with different Hg^2+^ concentrations; as the Hg^2+^ concentration increased from 7 to 70 ppm, the SPR of AgNPs sharply decreased. According to the corresponding photograph of the samples (Figure 9), AgNPs color changed from brown to colorless as Hg^2+^ concentration increased. This confirmed the AgNPs Hg^2+^ concentration‐dependent colorimetric activity and was consistent with the SPR spectra. Due to the direct correlation between the concentration of Hg^2+^ and the change in absorbance, AgNPs can be used as a colorimetric nanoprobe for quantitative Hg^2+^ detection. **Figure** **10** depicts the linear relationship between Hg^2+^ concentrations and the change in absorbance, which was calculated using the equation *y* = 0.0026x+0.0778 (*R* ^2^ = 0.965) (Hg^2+^ concentration ranged from 7 to 63 ppm). Equation (2) was used to calculate the limit of detection (LOD) of the AgNPs colorimetric probe to be 28 ppm within the Hg^2+^ range of 7–63 ppm. Equation (3) was used to calculate the limit of quantification (LOQ) of the colorimetric AgNPs probe at 85 ppm. A comparable outcome was also seen for AgNPs that was synthesized using phytochemicals,^[^ ^61^ ^]^ despite the LOD value being somewhat larger than the previously published reports.^[^ ^3^, ^42^, ^53^, ^60^, ^62^ ^]^ Hence, the quantification test of Hg^2+^ can make use of the colorimetric probe. Additionally, after 1 min, little discernible difference was observed, whereas, after 10 min, no discernible difference was seen (**Figure** **11**). This implied a potential use for the biosynthesized AgNPs for the unaltered quick visual colorimetric detection of Hg^2+^ ions with high sensitivity and significantly low limit of detection level.

Figure 9 explains the molecular underpinnings of the Hg^2+^ interaction with green synthesized AgNPs, which led to modifications in the absorption spectra and perturbations in the color observable to the necked eye. Various phytochemicals acting as stabilizers help explain why the prepared AgNPs are stable in an aqueous environment. AgNPs (Ag^0^) and Hg^2+^ will undergo a redox reaction as a result of the Hg^2+^ addition, which will speed up the process of Hg^2+^ attachment and adsorption to the negatively charged AgNPs surfaces. Different phytochemical stabilizers on the nanoparticles are damage in the procedure. As a result, Hg^2+^ will oxidize Ag^0^ to produce Hg^0^ and Ag^+^ while also reducing it. The gradual decrease of AgNPs color intensity upon Hg^2+^ addition is evidence of the produced AgNPs degradation or deterioration or disintegration. Similar phenomena were also described in different reports.^[^ ^3^, ^23^, ^56^ ^]^

## 3 Conclusion

In conclusion, this study reveals that green AgNPs, which were produced using *Piper chaba* stem extract, exhibit significant antibacterial activity against several gram‐positive and gram‐negative bacterial strains. They may therefore be employed as possible antibacterial agents in the medical and food industries. The greenly produced AgNPs also exhibit outstanding radical scavenging activity in the DPPH experiment with an estimated *IC*_50_ value of 1.13 mL (equivalent to 0.45 mg mL^−1^). The obtained AgNPs were successfully used for Hg^2+^ colorimetric detection in a solution for the first time. Without needing a laborious pretreatment process, the colorimetric responses can be seen with the naked eye and evaluated by UV–vis spectrophotometry. With a LOD of 28 ppm and a LOQ of 85 ppm, this colorimetric detection offers a straightforward, quick, and affordable method for measuring Hg^2+^ ions in an aqueous solution. This work is entirely green in light of the contributing materials employed, unlike some published detection strategies for Hg^2+^ where harmful compounds were used to create the nanomaterial. Thus, these outcomes of the green synthesized AgNPs may offer better quality materials for antibacterial, antioxidant as well as for sensing applications.

## 4 Experimental Section

### Materials

Silver nitrate (AgNO_3_) and 2, 2‐diphenyl‐1‐picrylhydrazine (DPPH) were purchased from Sigma‐Aldrich, Germany and Sigma Chemical Co. (St. Louis, MO), respectively. BaCl_2_·2H_2_O was purchased from BDH, UK. NiCl_2_·6H_2_O, CoCl_2_·6H_2_O, and CdSO_4_·8H_2_O were purchased from Loba Chemie, India. MgNO_3_·6H_2_O, PbNO_3_, and FeSO_4_·7H_2_O were purchased from Merck, India. HgCl_2_ was obtained from Molychem, India.

### Measurements

Ultraviolet‐visible (UV–vis) spectroscopic analysis was carried out on a SHIMADZU (Tokyo, Japan) UV‐1900i UV–vis spectrophotometer. Absorption spectra were recorded at a resolution of 1 nm within 200–800 nm. Transmission electron microscopy (TEM) measurement was conducted on a JEOL TEM‐2100F field emission electron microscope.

### Preparation of Silver Nanoparticles

The previous report stated that *Piper chaba* stem extract was used in the preparation of AgNPs.^[^ ^57^ ^]^ Briefly, in the typical procedure, 80 mL of freshly prepared 1 mm AgNO_3_ (aq.) solution was combined with 4 mL (20 g per 200 mL) of *Piper chaba* stem aqueous extract, and the reaction mixture was heated at 60 °C in an oil bath for 30 min while being constantly stirred. After centrifuging, ultrapure water was used to wash the resulting AgNPs. The obtained AgNPs were employed for antibacterial and antioxidant activity tests as well as for the colorimetric detection of the Hg^2+^ ions test.

### Antibacterial Activity Test

According to the reported literature, a well‐known disc diffusion method was used to investigate the assessment of bactericidal activity.^[^ ^63^, ^64^, ^65^ ^]^ In this assay, two gram‐positive strains [*Staphylococcus aureus* (ATCC 25923) and *Micrococcus Spp*.] and two gram‐negative strains [*E. coli* (ATCC 8739) and *Pseudomonas aeruginosa* (ATCC 27833)] of pathogenic bacteria were used. For 24 h at 37 °C, these bacterial cultures were revived in nutritional broth. The bacterial strains were grown on a nutrient agar medium. Whatman no. 1 filter paper was used to create 5 mm diameter filter paper discs. The assay's media, filter paper discs, and other equipment were autoclaved for sterilization, and the experiment was carried out in a microbiological safety cabinet. On nutrient agar plates that had hardened and been labeled, bacterial strains were streaked. The standard medication used was Eromycin DS. To assess the antibacterial activity, two different water dispersions of *Piper chaba*‐derived AgNPs (1.3 and 2.6 mg mL^−1^) were used. Each disk was loaded separately with 10 µL of each dispersion and 10 µL of Eromycin DS (1 mg mL^−1^). In a petri dish, the discs containing AgNPs were positioned in their proper locations and incubated for 24 h at 37 °C. After 24 h of incubation, the zone of inhibition was visible. The diameter of the inhibitory zone was measured to gauge the antibacterial activity. Three repetitions of the experiments were run, and the mean of the readings was recorded.

### Antioxidant Activity Test

A test for antioxidant activity was performed in accordance with earlier studies with a small modification.^[^ ^29^, ^66^ ^]^ AgNPs stock colloidal solution was first made. A stock solution with a 450 mg L^−1^ concentration was prepared. AgNPs solution in various concentrations of 0.1, 0.2, 0.3, 0.4, 0.5, 0.6, 0.7, 0.8, 0.9, 1.0, 1.1, 1.2, 1.3, 1.4, and 1.5 mL were taken in various test tubes. Each test tube received 0.5 mL of deionized water before being forcefully shaken. In each test tube, 2 mL of a 0.007 886% ethanolic solution of DPPH was then added. The mixture was forcefully swirled for 15 s. The solution was then let to stand at room temperature in a dark area for 30 min to allow for reaction. After 30 min, a UV–visible spectrophotometer was used to compare the absorbance to a control at 517 nm. Equation (1) was used to calculate the percentage of DPPH radical‐scavenging activity of green synthesized AgNPs

(1) $$\begin{array}{c} \mathrm{Percent}\;\mathrm{scavenging}\;\mathrm{activity}=\left(1-\frac{\mathrm{Absorbance}\;\mathrm{of}\;\mathrm{sample}\;}{\mathrm{Absorbance}\;\mathrm{of}\;\mathrm{blank}}\right)\;\times 100 \end{array}$$

The percentage of DPPH free radical‐scavenging activity was plotted against the amount of AgNPs to estimate the amount of AgNPs required to reduce DPPH radical‐scavenging by 50% (called *IC*_50_).

### Detection of Hg^2+^ Ions

A variety of metal ions stock solutions at various concentrations were made and AgNPs were added to the sample in an equal amount to serve as a colorimetric probe. The absorbance was then estimated using a UV–vis spectrophotometer, and the outcome was assessed using ultrapure water as a control sample. 2.5 mL of a solution containing 70 ppm of important metal ions, including Ba^2+^, Co^2+^, Cd^2+^, Fe^2+^, Hg^2+^, Mg^2+^, Ni^2+^, and Pb^2+^, were added to the detection system (1.5 mL of AgNPs) to determine the selectivity. AgNPs, Hg^2+^ (35 ppm), and interfering metals solutions (Ba^2+^, Co^2+^, Ni^2+^, Cd^2+^, Mg^2+^, Pb^2+^, and Fe^2+^) (35 ppm) were mixed in an equal proportion to simulate the effects of additional interfering metal ions. The mixture's total volume (4 mL) remained constant. Also, under the same circumstances, the AgNPs probe evaluated various quantities of Hg^2+^ samples (7–70 ppm) to assess the probe's accuracy. After mixing all of the test samples, they were to be held for 10 min before the absorption was measured. To determine the concentration of Hg^2+^, the measured absorption was transformed into a linear equation. Based on the following equation, the limit of detection (LOD) and limit of quantification (LOQ) of the Hg^2+^ colorimetric detection by the synthesized AgNPs were determined^[^ ^22^ ^]^

(2) $$\begin{array}{c} \mathrm{LOD}\;=\;\frac{3\;\times \;S_{i}}{b} \end{array}$$

(3) $$\begin{array}{c} \mathrm{LOQ}\;=\;\frac{10\;\times \;S_{i}}{b} \end{array}$$

where, *S*_i_ = standard deviation of the intercept and *b* = slope of the linear fitting.

## Conflict of Interest

The authors declare no conflict of interest.

## Data Availability

The data that support the findings of this study are available from the corresponding author upon reasonable request.
